# A Preliminary Study on the Modifying Effect of Strawberry Seed Oil and Sex on Rabbit Meat Quality

**DOI:** 10.3390/ani14223234

**Published:** 2024-11-12

**Authors:** Sylwia Ewa Pałka, Zuzanna Siudak, Michał Kmiecik, Agnieszka Otwinowska-Mindur, Małgorzata Grzesiak

**Affiliations:** 1 Department of Genetics, Animal Breeding and Ethology, University of Agriculture in Krakow, Al. Mickiewicza 24/28, 30-059 Kraków, Poland; michal.kmiecik@urk.edu.pl (M.K.); agnieszka.otwinowska@urk.edu.pl (A.O.-M.); 2 Department of Small Livestock Breeding, National Research Institute of Animal Production, Krakowska 1, 32-083 Balice, Poland; zuzanna.siudak@iz.edu.pl; 3 Department of Endocrinology, Faculty of Biology, Institute of Zoology and Biomedical Research, Jagiellonian University, Gronostajowa 9, 30-387 Kraków, Poland; m.e.grzesiak@uj.edu.pl

**Keywords:** strawberry seed oil, slaughter and meat quality traits, fatty acid profile, plasma lipid concentration, Termond white rabbits

## Abstract

The aim of this research was to determine the effect of strawberry seed oil addition to rabbit feed on slaughter performance traits, meat quality traits and plasma cholesterol and triglycerides of purebred Termond White rabbits (*n* = 24). The experiment used young rabbits born to 6 does. From each litter, two rabbits were randomly assigned into one of the two groups. In each group (2 × *n* = 12; 6♂, 6♀), the animals were fed a complete pelleted feed. Rabbits from the experimental group received feed enriched with a 1% addition of strawberry seed oil. Animals were slaughtered on day 84. Rabbits fed with pellets with the addition of strawberry oil were characterized by a higher hind part and head and liver weight compared to the rabbits from the control group (*p* ≤ 0.05). The addition of strawberry seed oil significantly increased the pH of matured meat and affected the colour coordinates of the meat by increasing their values (*p* ≤ 0.05). The tested oil reduced the values of meat texture parameters such as springiness, cohesiveness and chewiness (*p* ≤ 0.05). The tested oil impacted the level of HDL, which was higher in the experimental group, as well as lowered the triglyceride level. The addition of strawberry seed oil was proven to significantly increase the linoleic acid content in rabbit meat (*p* ≤ 0.05).

## Simple Summary

The seeds of strawberries are rich in polyenoic fatty acids (EUFAs) and fat-soluble vitamins such as A, D, E and K. Additionally, strawberry seed oil contains a high content of polyunsaturated fatty acids (PUFAs) in its composition. In addition, strawberry seed oil contains several bioactive substances, such as *p*-coumaric acid, ferulic acid, tyrosol, homovanillic acid, vanillic acid, vanillin and bioactive phenolic compounds. Therefore, we hypothesize that strawberry (*Fragaria × ananassa*) seed oil addition to rabbit feed influences slaughter performance traits and meat quality traits and improves plasma cholesterol and triglyceride levels in purebred Termond White rabbits. The experimental material involved 24 rabbits of the purebred Termond White breed (12♂, 12♀). The experiment used young rabbits born to 6 does. From each litter, two rabbits were randomly assigned into one of the two groups. In each group (2 × *n* = 12; 6♂, 6♀), the animals were fed a complete pelleted feed. Rabbits from the experimental group received feed enriched with a 1% addition of strawberry seed oil. Animals were slaughtered on day 84. Rabbits fed with pellets with the addition of strawberry oil were characterized by a higher hind part weight and head and liver weight compared to the rabbits from the control group (*p* ≤ 0.05). The addition of strawberry seed oil significantly increased the pH of matured meat and affected the colour coordinates of the meat by increasing their values (*p* ≤ 0.05). The tested oil reduced the values of meat texture parameters such as springiness, cohesiveness and chewiness (*p* ≤ 0.05). The tested oil impacted the level of HDL, which was higher in the experimental group, as well as lowered the triglyceride level. The addition of strawberry seed oil was proven to significantly increase the linoleic acid content in rabbit meat (*p* ≤ 0.05). The supplementation of strawberry seed oil does not affect the post-slaughter traits and meat quality of rabbits. In addition, supplementation with this oil might have a positive effect on the lipid profile in the blood of rabbits, namely, reducing triglycerides and increasing HDL content.

## 1. Introduction

The strawberry (*Fragaria* × *ananassa*) oil is produced from the seeds that are a by-product of the industrial processing of strawberries [1]. The seeds of strawberries are a rich source of polyenoic fatty acids (EUFAs) and fat-soluble vitamins such as A, D, E and K [2]. Additionally, strawberry seed oil contains a high content of polyunsaturated fatty acids (PUFAs) in its composition. Their level can be as high as 78% of the total fat content. These include α-linolenic acid, which can amount to as much as 42.22 g in 100 g of oil, and apart from this, they have an appropriate omega-6: omega-3 ratio content (*n*-6/*n*-3: 1.16 g/100 g) [3]. Polyunsaturated fatty acids have been found to reduce the risk of cardiovascular disease, lower blood pressure, have anticancer properties and enhance the immune system [4]. In addition, strawberry seed oil contains several bioactive substances, such as *p*-coumaric acid, ferulic acid, tyrosol, homovanillic acid, vanillic acid, vanillin and bioactive phenolic compounds. It has also been shown that strawberry oil is rich in tocopherols and tocotrienols, which are derivatives of vitamin E [1]. Tocopherols protect membrane phospholipids and preserve them from oxidation, which is important for structures with a high content of PUFAs and those exposed to oxygen. Vitamin E derivatives are used, among other things, in the treatment of habitual miscarriage, infertility and progressive muscular dystrophy or in the elimination of potency disorders. Vitamin E deficiency can lead to neurological disorders resulting from the peroxidation of cell membranes [5,6]. The vitamin E derivative α-tocopherol also has properties other than antioxidant. It inhibits protein kinase C (PKC), the growth of certain cells, including smooth muscle cells, and the transcription of CD36 and collagenase genes [7]. α-Tocopherol affecting protein kinase C inhibits platelet aggregation, nitric oxide production in endothelial cells, and superoxide production in neutrophils and macrophages [8].

Scientific studies confirm the beneficial effects of strawberry seed oil on the organism. A recent study showed that supplementation of 7% strawberry seed oil effectively lowers plasma triglyceride levels [9]. Also, the addition of blackcurrant, strawberry and raspberry seed oil reduced fat content in the livers of rats [10]. The aforementioned oils can improve oxidative stability, hence reducing oxidative stress. Szczurek-Janicka et al. [4] found that the addition of raspberry seed oil and strawberry seed oil, at 0.8 g per animal per day, which are sources of *n*-3 and *n*-6 PUFA, significantly increased the levels of alpha-linolenic acid (ALA *n*-3) and linoleic acid (LA *n*-6) in the muscles of the rats.

The possibility of rapidly improving the fatty acid profile of meat through supplementation with appropriate nutritional supplements has been confirmed in many scientific studies. Ferreira et al. [11] studied the effect of the addition of fish oil at 2.5 g/kg feed, 5 g/kg feed and 7.5 g/kg feed fed to lambs. There was an increase in conjugated linoleic acid (CLA) in the meat of lambs fed diets with added fish oil. In addition, the 7.5% addition of oil increased the content of eicosapentaenoic acid (EPA) and docosahexaenoic acid (DHA) in the lamb. Marounek et al. [12] fed rabbits from weaning on day 35 with a commercial CLA supplement. Animals received 5 g CLA/kg feed for 21 or 42 days and 10 g CLA/kg feed for 21 days before slaughter. The authors of the study considered a significant increase in PUFA in all experimental groups to be beneficial. The level of CLA in the tissues increased significantly with the increase in CLA in the diet. Pietras and Orczewska-Dudek [13] fed broiler chickens with 3% or 6% *Camelina sativa* oil. The addition of 6% *Camelina sativa* oil to the feed mixture significantly reduced the content of total cholesterol and its fractions in the blood plasma of the chickens compared to the other groups. The introduction of 3% and 6% *Camelina sativa* oil into the chickens’ diet enriched the breast meat in *n*-3 PUFAs, mainly α-linolenic acid (ALA). Oliveira et al. [14] fed bulls a 3.8% addition of soybean oil or linseed oil and commercial preparations of rumen-protected soybean oil and protected linseed oil at 4.5%. More CLA was found in meat from animals fed unprotected soybean oil, while better *n*-6/*n*-3 ratios were recorded in animals fed unprotected linseed oil. Morshedy et al. [15] fed rabbits with 0.3 mL/kg feed of rocket seed oil (*Eruca sativa Mill.*) and 0.3 mL/kg feed of wheat germ oil (*Triticum aestivum* L.), and their mixture of 0.15 mL of rocket seed oil and 0.15 mL of wheat germ oil in 1 kg of mixed feed. A higher content of linolenic acid in meat was observed in rabbits given rocket seed oil and wheat germ oil. Palmitic acid and oleic acid levels were reduced in the meat of animals fed with wheat germ oil and its mixture with rocket seed oil. Overall SFA levels were lowered in meat from rabbits fed a mixture of tested oils. According to the literature data, strawberry seed oil is a source of biologically valuable substances. The addition of strawberry seed oil to animal feed can have a beneficial effect not only on the functioning of the animal body but also on the quality of the obtained product—meat. Meat enriched with *n*-3 and *n*-6 PUFA, which can be considered functional food, is a unique product that enhances the human diet and can have a healthy effect on the human body.

Our previous studies on the use of raspberry seed oil in rabbit nutrition have shown that even a small addition (1%) of the aforementioned oil has a profitable effect on the fatty acid profile of meat and the reduction of cholesterol and triglycerides in rabbit plasma [16]. Therefore, we hypothesize that strawberry (*Fragaria* × *ananassa*) seed oil addition to rabbit feed influences slaughter performance traits and meat quality traits and improves plasma cholesterol and triglyceride levels in purebred Termond White rabbits.

## 2. Materials and Methods

### 2.1. Animals

The experiment was performed according to standardized conditions at the Experimental Station of the Department of Genetics, Animal Breeding and Ethology of the University of Agriculture in Kraków. In this study, 24 rabbits of the purebred Termond White breed (12 males and 12 females) were used. Until weaning at 35 days of age, young rabbits were kept in wooden cages standing in a hall equipped with water (nipple drinkers) and lighting (14 h of light) and forced ventilation. The average daily temperature in the hall was 12 °C. Next (days 35–84), animals were housed in metal cages designed for rabbit rearing. The single cage measured 40 × 30 × 35 cm (W × L × H). The experiment used young rabbits born to 6 does of the primary herd. A natural mating system was used. Mating was planned based on the CFC program [17]. All litters were born on the same day. Two rabbits from each litter were randomly allocated into one of the two groups. In each group (2 × *n* = 12; 6♂, 6♀), the animals were fed a complete pelleted feed (*ad libitum*) with constant access to drinking water. Rabbits from the experimental group (S) received feed enriched with a 1% addition of strawberry (*Fragaria* × *ananassa*) seed oil. Cold-pressed strawberry seed oil was bought from a feed manufacturer at an online shop and mixed with all the ingredients that were then pelleted. The animals were fed in groups (1 feeder per 2 animals of the same gender). The composition of the feed rations that the rabbits received in each group is presented in Table 1, while Table 2 shows the chemical composition. Analyses of the chemical composition of feed were carried out by the laboratory of the Department of Nutrition, Animal Biotechnology and Fisheries of the University of Agriculture in Kraków, Poland.

### 2.2. Carcass Traits

The animals were slaughtered on day 84 in a dedicated facility for the slaughter of animals at an average body weight of 2667 ± 212 g. The animals were stunned, immediately bled, pelted and eviscerated. Stunning was carried out using a mechanical method by means of a hard and precise blow causing brain damage, after which bleeding was induced, consequently leading to the rabbit’s immediate death. After slaughter, hot carcass weight (HCW) was recorded, and after 24 h of storage at 4 °C, chilled carcass weight (CCW) was also recorded. The carcass was then divided into three parts as follows: the fore part, the middle part, and the hind part, which were weighed. All measurements were made using electronic scales of Łucznik KS-205 (Galeria Łucznik Co., Ltd., Wrocław, Poland, e = 0.1). Post-slaughter traits included hot carcass weight (HCW), cold carcass weight (CCW); the weight of the fore part (FP), middle part (MP) and hind part (HP); head weight (HEAD) and liver weight (LIV). In addition, the hot (HDP) and cold dressing (CDP) percentages were counted according to the following formulas: HDP was defined as 100 × (HCW/SW), CPD = 100 × (CCW/SW) and SW in these equations means slaughter weight.

All procedures were performed according to the relevant guidelines and regulations. The authors confirm that the experiment complied with the European Union’s Directive on Animal Experimentation (Directive 2010/63/EU) and ARRIVE guidelines. The experiment was carried out after receiving approval from the 2nd Local Institutional Animal Care and Use Committee (IACUC) in Kraków (agreement no. 267/2018) and the Institutional Animal Care Review Board of the Faculty of Animal Sciences, University of Agriculture in Kraków permissions (approval numbers: 29/2016, 37/2016, and 3/2018).

### 2.3. pH and Colour Measurement and Fat Content Analysis

Meat colour in CIE colour space L*a*b* (L*—lightness, a*—redness and b*—yellowness), illuminant D65, measurement angle 0°, observer angle 2° and calibration against white [18] were measured 45 min and 24 h after slaughter on the surface of the muscles, namely, the longest lumbar muscle (*m. Longissimus lumborum*, at the level of the first lumbar vertebra) and biceps femoris (*m. Biceps femoris*, halfway down the muscle), always on the right side of the carcass. The final result for each parameter was the arithmetic mean of three measurements at different points over the surface of the *Longissimus lumborum* and *Biceps femoris* muscles. The pH of post-slaughter muscle tissue was measured using a microprocessor-based pH meter equipped with a SP24B-reinforced glass-driven electrode 45 min after slaughter (pH_45_) and 24 h after slaughter (pH_24_) with an accuracy of 0.01. Measurements were taken on the right side of the carcass of the same muscles and at the same locations as for meat colour. Meat pH was measured using a Hanna Instruments HI98163 pH meter (Hanna Instruments Co., Ltd., Bedfordshire, UK), while meat colour was measured using a Minolta CR-410 colorimeter (Minolta Co., Ltd., Osaka, Japan). The pH and colour of the meat were measured at room temperature (20°) in a room equipped with gravity ventilation and 150 lux of artificial lighting.

The fat content was determined on samples from *m. Longissimus lumborum* according to official methods of analysis of AOAC International, 18th edition, Method 991.36 Fat (Crude) In Meat and Meat Products.

### 2.4. Texture Analysis

Texture samples were cut using a GIESSER butcher knife by cutting the muscle attachments from the animal skeleton (the right side of the carcass). From the muscle prepared in that manner, a 2 cm long sample with a cylindrical cross-section was cut across the muscle fibres. The samples were packaged in rhombus-textured vacuum packaging bags made of PA/PE film with a thickness of 105 microns using a vacuum packer RCVG-32E (Royal Catering Co., Ltd., Berlin, Germany), freezing and then frozen for 72 h at −18 °C. After the stated time, the meat was thawed at room temperature and then cooked for 40 min in a water bath set at 80 °C [19]. Shear force and texture profile analysis parameters (hardness, chewiness, cohesiveness and springiness) were measured using a TA.XT plus texturometer (Stable Micro Systems Co., Ltd., Godalming, UK). Shear force was measured on cubic specimens using a Warner-Bratzler blade (Stable Micro Systems Co., Ltd., Godalming, UK) with a triangular notch. Specimens with a cross-sectional area of 10 × 10 mm were cut perpendicular to the course of the muscle fibres at a blade speed of 2 mm/s. Profile texture analysis was measured using a 50 mm diameter cylinder attachment. A double compression test was performed on cubic specimens up to 75% of the specimen height [19]. The speed of the roller was 5 mm/s, and the interval between pressures was 5 s. All results were counted automatically using Exponent for Windows version 5.1.2.0 (Stable Micro Systems Co., Ltd., Godalming, UK), which is compatible with the texturometer software.

### 2.5. Drip Loss

Samples (*m. Longissimus lumborum*) of approximately 50 g were tightly packed in ziplock bags. The material thus prepared was placed at 75 °C for 50 min in a water bath. After pasteurization, samples were cooled at room temperature, dried and weighed to the nearest 0.01. The difference between sample weight before and after boiling divided by sample weight before boiling was expressed as a percentage.

### 2.6. Fatty Acids Analysis

Meat samples were extracted with a chloroform-methanol solution according to the method of Folch et al. [20]. Fatty acid methyl esters were prepared according to ISO 12966-2:2011. The fatty acid profile of the corresponding methyl esters was determined by gas chromatography using a Trace GC Ultra gas chromatograph (Thermo Electron Corp., Waltham, MA, USA) equipped with a 30 m capillary column (Supelcowax, Bellefonte, PA, USA) with an inner diameter of 0.25 mm and a coating thickness of 0.25 µm. Helium with a flow rate of 1 mL/min was used as carrier gas. The dispenser and detector temperatures were 220 °C and 250 °C, respectively. The column temperature was initially 160 °C for 3 min and then increased by 3 °C every minute until a temperature of 210 °C was reached, which was maintained for 25 min.

### 2.7. Lipid Profile Analysis

Blood samples collected into heparinized tubes at the time of slaughter were centrifuged at 4000× *g* for 10 min. Separated plasma fractions were snap-frozen in liquid nitrogen for further lipid profile analysis. Plasma triglycerides, total cholesterol and its high-density fraction (HDL) levels were assayed with commercial colorimetric kits (cat. No. T7532, C7510 and H7511, respectively; Pointe Scientific, Brussels, Belgium) following the manufacturer’s instruction. The content of the low-density fraction of cholesterol (LDL) was estimated according to the formula: LDL = total cholesterol—HDL—(triglycerides/5). Detection limits were 5 mg/dL for triglycerides, 3 mg/dL for total cholesterol and 3 mg/dL for HDL. The intra-assay and inter-assay coefficients of variation for triglycerides, total cholesterol and HDL were 1.62% and 1.92%, 1.32% and 1.93%, 4.7% and 5.1%, respectively. All analyses were performed in duplicate.

### 2.8. Statistical Analysis of the Results

The following linear model was fitted using the MIXED procedure in SAS [21] as follows:

Y_ijk_ = µ + Feed_i_ + Sex_j_ + (Feed × Sex)_ij_ + e_ijk_

where

Y_ijk_—post-slaughter traits or meat quality parameter.

µ—overall mean.

Feed_i_—fixed effect of *i*-th feeding (*i* = 1, 2).

Sex_j_—fixed effect of *j*-th sex (*j* = 1, 2).

(Feed × Sex)_ij_—fixed effect of interaction between feeding and sex.

e_ijk_—residual effect.

The significance of differences between least square means was determined by the Tukey–Kramer test. All *p*-values less than or equal to 0.05 (*p* ≤ 0.05) were considered significant.

## 3. Results

### 3.1. Carcass Traits

Table 3 shows the effect of feeding and sex on post-slaughter traits. Rabbits fed with pellets with the addition of strawberry oil were characterized by a higher hind part weight, head weight and liver weight compared to the rabbits from the control group (*p* ≤ 0.05). There were no significant differences in the pre-slaughter body weight, hot and cold carcass weight, fore part and middle part and hot and cold dressing out percentage between the food groups studied (Table 3). The female carcasses were characterized by a significantly higher (*p* ≤ 0.05) pre-slaughter weight, hot and cold weight, fore, middle and hind part weight and liver weight compared to male carcasses. There were no significant differences between the sexes for other post-mortem traits (*p* > 0.05; Table 3).

### 3.2. Acidity and Colour and Fat Content

The second studied group of traits was meat quality traits. The least squares means (LSMs) of pH and colour measurement traits are presented in Table 4. It was found that the pH measured after 24 h on the *longissimus lumborum* muscle and the *biceps femoris* muscle differed significantly (*p* ≤ 0.05) between groups, with meat obtained from the experimental group (S) having a higher value for this parameter. Meat (*longissimus lumborum* muscle) obtained from males had a higher value for pH_24_ (*p* ≤ 0.05; Table 4). For most colour measurement traits, the differences depending on the feeding regimes had been significant (*p* ≤ 0.05), except the lightness (L*_45_) and redness (b*_24_) on the *m. Biceps femoris* and lightness (L*_24_) on the *m. Longissimus lumborum*. The carcasses of the rabbits from the control group were characterized by significantly higher (*p* ≤ 0.05) a* and b* values measured 45 min after the slaughter on the *m. Biceps femoris* and *m. longissimus lumborum* and 24 h after the slaughter on the *m. Longissimus lumborum.* The higher value (*p* ≤ 0.05) of lightness (L*_24_) and yellowness (b*_24_) on the *m. Biceps femoris* and lightness (L*_45_) on the *m. Longissimus lumborum* was observed in the experimental group in comparison with the control group (Table 4). Sex also significantly affected a*_45_ of the *Biceps femoris* muscle, and significantly higher values were obtained for meat from males (*p* ≤ 0.05). In contrast, significantly higher lightness (L*_45_) of the *Longissimus lumborum* muscle 45 min after slaughter was found in females (*p* ≤ 0.05; Table 4). The addition of strawberry seed oil was not shown to significantly affect the fat content of the rabbits’ *longissimus lumborum* muscle (Table 4).

### 3.3. Drip Loss and Meat Texture

The addition of strawberry seed oil, as well as sex, had a significant effect (*p* ≤ 0.05) on rabbit meat springiness and chewiness. In addition, the diet also had a significant effect (*p* ≤ 0.05) on meat cohesiveness. Animals in the control group had higher values for these parameters compared to animals in the experimental group. The meat of females was characterized by higher springiness and chewiness compared to that of males (*p* ≤ 0.05; Table 5).

### 3.4. Texture Analysis

The addition of strawberry seed oil, as well as sex, had a significant effect (*p* ≤ 0.05) on rabbit meat springiness and chewiness. In addition, the diet also had a significant effect (*p* ≤ 0.05) on meat cohesiveness. Animals in the control group had higher values for these parameters compared to animals in the experimental group. The meat of females was characterized by higher springiness and chewiness compared to that of males (*p* ≤ 0.05; Table 5).

### 3.5. Plasma Cholesterol and Triglycerides

The analysis carried out showed that the addition of strawberry seed oil influenced the level of triglycerides (*p* ≤ 0.05), which was diminished in comparison to the control group. However, the total cholesterol level did not change following the addition of strawberry seed oil. The addition of strawberry seed oil impacted the level of HDL (*p* ≤ 0.05), which was higher in the experimental group. LDL level was similar in the two groups. Sex did not significantly affect blood cholesterol, HDL, LDL and triglyceride levels in rabbits (Table 6).

### 3.6. Fatty Acid Profile

The supplementation of strawberry seed oil had a marked effect (*p* ≤ 0.05) on the fatty acid profile of the meat (*m. longissimus lumborum*), as shown in Table 7. Animals in the experimental group had higher fatty acid content of 12:0, 14:0, 18:2 *n*-6 and lower fatty acid content of 16:0, 18:1 *n*-7, 20:3 *n*-6, 20:5 *n*-3, 22:4 *n*-6 and 22:5 *n*-3 compared to animals in the control group (*p* ≤ 0.05). The analysis also showed that sex significantly affected (*p* ≤ 0.05) the fatty acid level in rabbit meat. Meat from males was characterized by higher fatty acid content of 16:0 and 20:1 and lower fatty acid content of 14:1, 15:0, 16:1 *n*-9, 17:0, 18:1 *n*-9 and conjugated linoleic acids (CLAs) compared to meat from females (*p* ≤ 0.05; Table 7).

## 4. Discussion

The current study showed that animals receiving feed with strawberry oil had higher hind part, head and liver weights; lower meat acidity 24 h after slaughter; lower meat springiness, cohesiveness, and chewiness; reduced plasma triglyceride content and greater HDL fraction; and altered content of certain fatty acids compared to control animals. Herein, the addition of strawberry seed oil also increased the linoleic acid content and decreased the palmitic acid level; the effect of strawberry seed oil on the pH and colour of the rabbit meat was also noted. This effect was not only entirely positive as it would increase the pH of the meat but also made the carcasses brighter in colour than those obtained from animals in the control group.

The increased content of linoleic acid in the meat of rabbits treated with strawberry seed oil results in an improvement in its biological value because linoleic acid is an essential nutrient that cannot be synthesized by the human body and therefore must be supplied with food. As an essential component of ceramides, linoleic acid is involved in the maintenance of the transdermal epidermal water barrier, and its deficiencies in the diet of infants can result in skin lesions such as psoriasis, growth retardation and thrombocytopenia [22]. Linoleic acid in rabbit meat is also found in the form of conjugated linoleic acid (CLA), which has antioxidant, antiatherosclerotic and antidiabetic properties protecting the immune system and is involved in bone formation [23]. CLA increased content in the meat of oil-supplemented rabbits was also reported in our experiment, but the differences between groups were not significant.

Similar to strawberry seed oil, sunflower oil is also rich in *n*-6 fatty acids. Zsédely et al. [24] investigated the impact of rabbit feed supplemented with a mixture of two oils at a rate of 2% each: linseed oil as a rich source of α-linolenic acid belonging to the *n*-3 family and sunflower oil, containing mainly acids from the *n*-6 family, on the fatty acid composition of thigh and loin muscles. The results of the chemical analysis showed that the supplementation of feed with plant oils increased significantly the amount of linoleic and α-linolenic acids, reducing the proportion of SFA and MUFA in both muscles studied. There was also a significantly lower PUFA *n*-6/*n*-3 ratio and significantly higher PUFA/SFA ratio compared to the control group. Another oil rich in *n*-6 fatty acids is soybean oil. Alves dos Santos et al. [25], in an experiment using 1.5% and 3% addition of soybean oil, noted an improvement in the ratio of saturated to unsaturated fatty acids and an increased content of linoleic acid in rabbit meat.

Our study showed that female carcasses were characterized by a significantly higher pre-slaughter weight compared to male carcasses. Sex also significantly affected a*_45_ of the *biceps femoris* muscle, springiness and chewiness. Meat from males was characterized by higher 16:0, 20:1 fatty acid content and lower 14:1, 15:0, 16:1 *n*-9, 17:0, 18:1 *n*-9 and conjugated linoleic acids (CLAs) fatty acid content in comparison to meat from females.

The effect of gender on the fatty acid profile of rabbit meat was studied by North et al. [26]. The researchers performed meat quality analysis on the *m. longissimus lumborum* and *m. biceps femoris*, and the results indicated that gender influences the fatty acid profile of the muscles studied. Gender was shown to affect the oleic acid (18:1) content measured on the loin and hind leg, with higher oleic acid content in meat from males. In addition, a higher content of monounsaturated fatty acids (MUFAs) was noted in males. In females, the level of pentadecanoic acid (15:0) was significantly higher. Our present results have also proven that the level of pentadecanoic acid was higher in females. On the other hand, oleic acid content was lower in males.

The experiment on rabbits revealed that strawberry seed oil has a significant effect on the post-slaughter traits and quality of rabbit meat. Its effect on hind part weight, head weight and liver weight was found. The analysis indicates that strawberry seed oil significantly influences the acidity of meat, increasing its level but not enough to reduce meat quality and shelf life in the process. The nutritional additive also affects the colour of the meat, including its lightness. The addition of oil makes the meat lighter, which is not necessarily a defect. Along with this, the supplement lowers the values of meat texture profile analysis parameters such as springiness, cohesiveness and chewiness. Rich in omega-6 fatty acids, strawberry seed oil also significantly increased linoleic acid levels in the meat.

It is generally known that strawberry seed oil has a beneficial effect on the animal organism. The experiment presented here confirms this effect, as evidenced by the results of plasma triglyceride and HDL levels in rabbits. The addition of strawberry seed oil significantly decreased triglyceride and increased HDL level, which may help to reduce the incidence of diseases such as pancreatitis, hypertension, and atherosclerosis. Similar results were presented by Jurgoński et al. [9], who revealed diminished triglyceride concentration in rats. However, another study on rats did not show any effect of strawberry seed oil on lipid profile [22] that could result from used doses. Strawberry seed oil can thus not only be used as an additive to animal feed but also find use in human nutrition as a dietary supplement to help prevent cardiovascular disease.

## 5. Conclusions

Based on the above-mentioned findings, it can be concluded that the addition of strawberry seed oil does not affect the post-slaughter traits and meat quality of rabbits (excluding colour parameters) of both sexes. In addition, supplementation with this oil might have a positive effect on the lipid profile in the blood of rabbits, namely, reducing triglycerides and increasing HDL content.

## Figures and Tables

**Table 1:** Component composition of feed mixtures fed to rabbits in the control (C) and experimental groups (S), according to the composition provided by FHP Barbara Sp. z o.o. Feed Factory (Turza, Poland).

Component	Feeding ^1^	
	C	S
	Share of component (%)	
Wheat	29.58	28.58
Maize	24.50	24.50
Bran	15.00	15.00
Sunflower meal ^2^	11.00	11.00
Lucerne meal ^3^	10.00	10.00
Soyabean meal ^4^	7.00	7.00
Mineral and vitamin premix	1.50	1.50
Calcium carbonate	0.80	0.80
Dicalcium phosphate	0.62	0.62
Strawberry seed oil	0.00	1.00

^1^ C—control group, S—feed with 1% strawberry (*Fragaria × ananassa*) seed oil added. Each treatment (diet) contains (an amount per kilogram): vitamin A 10.000 IU; vitamin D3 1.500 IU; vitamin E 30 mg; Cu 7.5 mg; Fe 50 mg; Mn 75 mg; Zn 50 mg; I 1.0 mg; Se 0.2 mg. Crude protein: ^2^ 33%, ^3^ 16.4%, ^4^ 44%.

**Table 2:** Chemical composition of feed mixtures fed to rabbits in the control (C) and experimental groups (S).

Component	Feeding ^1^		SSO ^2^
	C	S	
	Content (g/kg)		
Dry matter	972.5	972.2	
Crude ash	74.0	71.4	
Total protein	160.4	157.0	
Crude fat	40.5	42.0	
Crude fibre	181.8	180.2	
Fatty acid:			
10:0	7.12	4.10	0.00
12:0	104.85	59.31	0.00
14:0	38.83	20.08	0.96
14:1	0.28	0.43	0.00
15:0	1.23	0.90	0.28
16:0	128.76	119.12	64.98
16:1 *n*-9	0.70	1.03	1.10
16:1 *n*-7	2.64	1.79	1.39
17:0	0.90	0.88	0.41
17:1	0.63	0.38	0.29
18:0	34.46	30.98	29.83
18:1 *n*-9	298.10	308.60	207.62
18:1 *n*-7	10.99	9.86	4.83
18:2 *n*-6	329.19	410.46	581.45
18:3 *n*-6	1.30	0.12	0.11
18:3 *n*-3	34.02	25.33	102.03
20:0	2.98	3.29	4.24
20:1	4.20	3.37	1.50

^1^ C—control group, S—feed with 1% strawberry (*Fragaria × ananassa*) seed oil added, ^2^ SSO—strawberry seed oil.

**Table 3:** Effect of feeding and sex on pre-slaughter weight and slaughter performance traits.

Parameter ^1^	Feeding ^2^				Sex				*p*-Value		
	C (*n* = 12)		S (*n* = 12)		♂ (*n* = 12)		♀ (*n* = 12)		Feeding	Sex	F*S ^5^
	LSM ^3^	SE ^4^	LSM ^3^	SE ^4^	LSM ^3^	SE ^4^	LSM ^3^	SE ^4^			
SW (g)	2612.50	41.37	2720.83	41.37	2520.83 ^a^	41.37	2812.50 ^b^	41.37	0.079	<0.001	0.169
HCW (g)	1417.25	27.56	1467.00	27.56	1372.83 ^a^	27.56	1511.42 ^b^	27.56	0.216	0.002	0.776
CCW (g)	1352.83	27.01	1410.42	27.01	1318.50 ^a^	27.01	1444.75 ^b^	27.01	0.147	0.003	0.755
FP (g)	560.67	11.22	588.33	11.22	547.92 ^a^	11.22	601.08 ^b^	11.22	0.096	0.003	0.926
MP (g)	300.17	9.11	300.83	9.11	285.42 ^a^	9.11	315.58 ^b^	9.11	0.959	0.029	0.789
HP (g)	492.00 ^a^	8.96	521.25 ^b^	8.96	485.17 ^a^	8.96	528.08 ^b^	8.96	0.032	0.003	0.583
HEAD (g)	129.17 ^a^	2.46	142.92 ^b^	2.46	134.25	2.46	137.83	2.46	0.001	0.315	0.759
LIV (g)	64.67 ^a^	3.06	80.42 ^b^	3.06	67.00 ^a^	3.06	78.08 ^b^	3.06	0.002	0.018	0.076
HDP (%)	54.30	0.50	53.91	0.50	54.51	0.50	53.70	0.50	0.578	0.263	0.068
CDP (%)	51.84	0.50	51.83	0.50	52.34	0.50	51.33	0.50	0.985	0.167	0.095
SW (g)	2612.50	41.37	2720.83	41.37	2520.83 ^a^	41.37	2812.50 ^b^	41.37	0.079	<0.001	0.169

^1^ SW—pre-slaughter body weight, HCW—hot carcass weight, CCW—cold carcass weight, FP—fore part, MP—middle part, HP—hind part, HEAD—head weight, LIV—liver weight, HDP—hot dressing out percentage, CDP—cold dressing out percentage, ^2^ C—commercial pelleted feed, S—feed with 1% strawberry (*Fragaria × ananassa*) seed oil added, ^3^ LSM—least square mean, ^4^ SE—standard error, ^5^ F*S—interaction between feed and sex, ^a, b^—averages in rows marked with different letters within feeding or sex are significantly different (*p* ≤ 0.05).

**Table 4:** Effect of feeding and sex on meat acidity, colour and fat content.

Parameter	Feeding ^1^				Sex				*p*-Value		
	C (*n* = 12)		S (*n* = 12)		♂ (*n* = 12)		♀ (*n* = 12)		Feeding	Sex	F*S ^4^
	LSM ^2^	SE ^3^	LSM ^2^	SE ^3^	LSM ^2^	SE ^3^	LSM ^2^	SE ^3^			
*Biceps femoris* muscle											
pH_45_	6.24	0.05	6.30	0.05	6.24	0.05	5.38	0.05	0.422	0.328	0.060
pH_24_	5.27 ^a^	0.03	5.43 ^b^	0.03	6.30	0.03	5.32	0.03	0.004	0.191	0.117
L*_45_	55.93	0.85	58.08	0.85	59.38	0.85	54.64	0.85	0.089	0.001	0.002
A*_45_	4.79 ^a^	0.39	2.73 ^b^	0.39	4.56 ^a^	0.39	2.96 ^b^	0.39	0.001	0.008	0.016
b*_45_	0.97 ^a^	0.58	−1.99 ^b^	0.58	−0.10	0.58	−0.91	0.58	0.002	0.334	0.593
L*_24_	55.49 ^a^	0.46	61.49 ^b^	0.46	58.15	0.46	58.82	0.46	<0.001	0.318	0.252
a*_24_	3.74	0.26	4.07	0.26	3.92	0.26	3.88	0.26	0.379	0.901	0.907
b*_24_	2.45 ^a^	0.25	3.82 ^b^	0.25	2.96	0.25	3.30	0.25	0.001	0.349	0.737
*Longissimus lumborum* muscle											
pH_45_	6.26	0.05	6.36	0.05	6.25	0.05	6.37	0.05	0.155	0.115	0.702
pH_24_	5.09 ^a^	0.03	5.31 ^b^	0.03	5.26 ^a^	0.03	5.13 ^b^	0.03	<0.001	0.002	0.138
L*_45_	55.42 ^a^	0.93	68.46 ^b^	0.93	58.98 ^a^	0.93	64.90 ^b^	0.93	<0.001	0.001	0.351
a*_45_	5.23 ^a^	0.64	1.67 ^b^	0.64	3.06	0.64	3.85	0.64	0.001	0.395	0.002
b*_45_	1.23 ^a^	0.89	−5.01 ^b^	0.89	−2.22	0.89	−1.56	0.89	<0.001	0.604	0.090
L*_24_	56.54	0.64	57.70	0.64	56.58	0.64	57.66	0.64	0.219	0.248	0.202
a*_24_	9.50 ^a^	0.61	6.97 ^b^	0.61	8.58	0.61	7.89	0.61	0.008	0.439	0.089
b*_24_	5.97 ^a^	0.40	3.02 ^b^	0.40	4.08	0.40	4.91	0.40	<0.001	0.151	0.111
Fat (%)	2.39	0.60	2.47	0.60	2.40	0.49	2.47	0.49	0.190	0.052	0.052

^1^ C—commercial pelleted feed, S—feed with 1% strawberry (*Fragaria x ananassa*) seed oil added, ^2^ LSM—least square mean, ^3^ SE—standard error, ^4^ F*S—interaction between feed and sex, ^a, b^—averages in rows marked with different letters within feeding or sex are significantly different (*p* ≤ 0.05).

**Table 5:** Effect of feeding and sex on drip loss, shear force and profile texture analysis of rabbit meat.

Parameter	Feeding ^1^				Sex				*p*-Value		
	C (*n* = 12)		S (*n* = 12)		♂ (*n* = 12)		♀ (*n* = 12)		Feeding	Sex	F*S ^4^
	LSM ^2^	SE ^3^	LSM ^2^	SE ^3^	LSM ^2^	SE ^3^	LSM ^2^	SE ^3^			
Drip loss (%)	26.89	0.73	24.89	0.73	26.07	0.73	25.71	0.73	0.067	0.738	0.052
Shear force (kg)	1.58	0.13	1.69	0.13	1.50	0.13	1.76	0.13	0.523	0.163	0.942
Hardness (kg)	12.39	0.52	11.39	0.52	11.22	0.52	12.56	0.52	0.189	0.084	0.147
Springiness	0.57 ^a^	0.02	0.50 ^b^	0.02	0.50 ^a^	0.02	0.56 ^b^	0.02	0.017	0.021	0.025
Cohesiveness	0.47 ^a^	0.01	0.44 ^b^	0.01	0.42	0.01	0.49	0.01	0.132	0.001	0.239
Chewiness (kg)	3.45 ^a^	0.28	2.53 ^b^	0.28	2.41 ^a^	0.28	3.56 ^b^	0.28	0.028	0.008	0.176

^1^ C—commercial pelleted feed, S—feed with 1% strawberry (*Fragaria × ananassa*) seed oil added, ^2^ LSM—least square mean, ^3^ SE—standard error, ^4^ F*S—interaction between feed and sex, ^a, b^—averages in rows marked with different letters within feeding or sex are significantly different (*p* ≤ 0.05).

**Table 6:** Effect of feeding and sex on plasma cholesterol and triglyceride levels.

Parameter (mg/dL)	Feeding ^1^				Sex				*p*-Value		
	C (*n* = 12)		S (*n* = 12)		♂ (*n* = 12)		♀ (*n* = 12)		Feeding	Sex	F*S ^4^
	LSM ^2^	SE ^3^	LSM ^2^	SE ^3^	LSM ^2^	SE ^3^	LSM ^2^	SE ^3^			
Cholesterol	2220.75	105.03	2100.92	105.03	2147.00	105.03	2174.67	105.03	0.429	0.854	0.110
HDL	131.29 ^a^	2.41	170.41 ^b^	2.41	154.03	2.41	147.66	2.41	<0.001	0.076	0.299
LDL	1956.86	103.57	1823.46	103.57	1874.67	103.57	1905.65	103.57	0.373	0.385	0.109
Triglycerides	663.00 ^a^	25.85	535.25 ^b^	25.85	591.50	25.85	606.75	25.85	0.002	0.681	0.381

^1^ C—commercial pelleted feed, S—feed with 1% strawberry (*Fragaria × ananassa*) seed oil added, ^2^ LSM—least square mean, ^3^ SE—standard error, ^4^ F*S—interaction between feed and sex, ^a, b^—averages in rows marked with different letters within feeding or sex are significantly different (*p* ≤ 0.05).

**Table 7:** Effect of feeding and sex on the fatty acid profile of rabbit meat.

Parameter (mg/dL)	Feeding ^1^				Sex				*p*-Value		
	C (*n* = 12)		S (*n* = 12)		♂ (*n* = 12)		♀ (*n* = 12)		Feeding	Sex	F*S ^4^
	LSM ^2^	SE ^3^	LSM ^2^	SE ^3^	LSM ^2^	SE ^3^	LSM ^2^	SE ^3^			
10:0	0.06	0.01	0.08	0.01	0.08	0.01	0.07	0.01	0.138	0.622	0.046
12:0	0.59 ^a^	0.06	0.88 ^b^	0.06	0.82	0.06	0.65	0.06	0.002	0.051	0.038
14:0	2.47 ^a^	0.13	3.33 ^b^	0.13	2.86	0.13	2.94	0.13	0.001	0.619	0.453
14:1	0.12	0.02	0.16	0.02	0.10 ^a^	0.02	0.17 ^b^	0.02	0.259	0.045	0.212
15:0	0.48	0.01	0.50	0.01	0.47 ^a^	0.01	0.51 ^b^	0.01	0.194	0.029	0.062
16:0	25.62 ^a^	0.44	23.87 ^b^	0.44	26.12 ^a^	0.44	23.38 ^b^	0.44	0.011	0.001	0.039
16:1 *n*-9	0.48	0.01	0.47	0.01	0.45 ^a^	0.02	0.49 ^b^	0.02	0.470	0.041	0.615
16:1 *n*-7	1.98	0.25	2.21	0.25	1.89	0.25	2.30	0.25	0.509	0.255	0.095
17:0	0.54	0.02	0.52	0.02	0.50 ^a^	0.02	0.56 ^b^	0.02	0.614	0.047	0.915
17:1	0.24	0.01	0.24	0.01	0.22 ^a^	0.01	0.26 ^b^	0.01	0.902	0.021	0.153
18:0	6.21	0.25	6.15	0.25	6.02	0.25	6.33	0.25	0.874	0.403	0.644
18:1 *n*-9	28.47	0.57	27.44	0.57	27.06 ^a^	0.57	28.86 ^b^	0.57	0.214	0.036	0.016
18:1 *n*-7	1.31 ^a^	0.03	1.14 ^b^	0.03	1.23	0.03	1.22	0.03	0.002	0.960	0.209
18:2 *n*-6	23.68 ^a^	0.74	26.73 ^b^	0.74	25.16	0.74	25.26	0.74	0.009	0.923	0.032
18:3 *n*-6	0.06	0.01	0.06	0.01	0.06	0.01	0.06	0.01	0.355	0.549	0.028
18:3 *n*-3	1.52	0.07	1.45	0.07	1.51	0.07	1.46	0.07	0.535	0.606	0.009
CLA	0.03	0.01	0.03	0.01	0.03 ^a^	0.01	0.04 ^b^	0.01	0.142	0.013	0.024
20:0	0.10	0.01	0.11	0.01	0.11	0.01	0.10	0.01	0.787	0.427	0.169
20:1	0.35	0.02	0.33	0.02	0.37 ^a^	0.02	0.31 ^b^	0.02	0.449	0.019	0.212
20:2	0.28	0.01	028	0.01	0.29	0.01	0.26	0.01	0.897	0.114	0.006
20:3 *n*-6	0.29 ^a^	0.02	0.18 ^b^	0.02	0.24	0.02	0.23	0.02	0.002	0.866	0.007
20:4 *n*-6	3.18	0.29	2.44	0.29	2.78	0.29	2.84	0.29	0.096	0.879	0.080
20:4 *n*-3	0.04	0.01	0.03	0.01	0.04	0.01	0.03	0.01	0.114	0.253	0.020
20:5 *n*-3	0.06 ^a^	0.01	0.03 ^b^	0.01	0.05	0.01	0.04	0.01	0.001	0.582	0.015
22:4 *n*-6	0.96 ^a^	0.08	0.67 ^b^	0.08	0.83	0.08	0.80	0.08	0.018	0.842	0.007
22:5 *n*-6	0.38	0.04	0.08	0.04	0.31	0.04	0.35	0.04	0.068	0.489	0.034
22:5 *n*-3	0.37 ^a^	0.03	0.27 ^b^	0.03	0.32	0.03	0.33	0.03	0.046	0.886	0.156
22:6 *n*-3	0.09	0.01	0.07	0.01	0.08	0.01	0.08	0.01	0.155	0.989	0.099
Total	99.97	0.01	99.99	0.01	99.98	0.01	99.98	0.01	<0.001	0.206	0.054

^1^ C—commercial pelleted feed, S—feed with 1% strawberry (*Fragaria × ananassa*) seed oil added, ^2^ LSM—least square mean, ^3^ SE—standard error, ^4^ F*S—interaction between feed and sex, ^a, b^—averages in rows marked with different letters within feeding or sex are significantly different (*p* ≤ 0.05).

## Data Availability

Data are contained within the article.
